# Tempol Reverses the Negative Effects of Morphine on Arterial Blood-Gas Chemistry and Tissue Oxygen Saturation in Freely-Moving Rats

**DOI:** 10.3389/fphar.2021.749084

**Published:** 2021-09-22

**Authors:** Santhosh M. Baby, Joseph F. Discala, Ryan Gruber, Paulina M. Getsy, Feixiong Cheng, Derek S. Damron, Stephen J. Lewis

**Affiliations:** ^1^ Galleon Pharmaceuticals Inc, Horsham, PA, United states; ^2^ Department of Pediatrics, Case Western Reserve University, Cleveland, OH, United states; ^3^ Genomic Medicine Institute, Lerner Research Institute, Cleveland Clinic, Cleveland, OH, United states; ^4^ Department of Biological Sciences, School of Biomedical Sciences, Kent State University, Kent, OH, United states; ^5^ Department of Pharmacology, Case Western Reserve University, Cleveland, OH, United states

**Keywords:** tempol, morphine, blood-gas chemistry, arterial blood pressure, tissue oxygen saturation, rats

## Abstract

We have reported that pretreatment with the clinically approved superoxide dismutase mimetic, Tempol (4-hydroxy-2,2,6,6-tetramethylpiperidine-N-oxyl), blunts the cardiorespiratory depressant responses elicited by a subsequent injection of fentanyl, in halothane-anesthetized rats. The objective of the present study was to determine whether Tempol is able to reverse the effects of morphine on arterial blood-gas (ABG) chemistry in freely-moving Sprague Dawley rats. The intravenous injection of morphine (10 mg/kg) elicited substantial decreases in pH, pO_2_ and sO_2_ that were accompanied by substantial increases in pCO_2_ and Alveolar-arterial gradient, which results in diminished gas-exchange within the lungs. Intravenous injection of a 60 mg/kg dose of Tempol 15 min after the injection of morphine caused minor improvements in pO_2_ and pCO_2_ but not in other ABG parameters. In contrast, the 100 mg/kg dose of Tempol caused an immediate and sustained reversal of the negative effects of morphine on arterial blood pH, pCO_2_, pO_2_, sO_2_ and Alveolar-arterial gradient. In other rats, we used pulse oximetry to determine that the 100 mg/kg dose of Tempol, but not the 60 mg/kg dose elicited a rapid and sustained reversal of the negative effects of morphine (10 mg/kg, IV) on tissue O_2_ saturation (SpO_2_). The injection of morphine caused a relatively minor fall in mean arterial blood pressure that was somewhat exacerbated by Tempol. These findings demonstrate that Tempol can reverse the negative effects of morphine on ABG chemistry in freely-moving rats paving the way of structure-activity and mechanisms of action studies with the host of Tempol analogues that are commercially available.

## Introduction

Morphine is a widely administered and effective analgesic agent. However, along with this positive effect, morphine also depresses breathing and impairs gas-exchange in the lungs, which combine to produce negative effects on arterial blood-gas (ABG) chemistry (Dahan et al., 2010; Boom et al., 2012; Henderson et al., 2014; Baby et al., 2018). These effects of morphine–analgesia and the depression of breathing–can be prevented or reversed by administration of opioid receptor antagonists, such as naloxone (Dahan et al., 2010). Nevertheless, the development of drugs that overcome the negative effects of opioids on breathing without affecting analgesia is an important effort because of numerous scenarios when administration of opioid receptor antagonists are not tenable or optimal (van der Schier et al., 2014; Dahan et al., 2018). For instance, a patient struggling to breathe due to the effects of opioids given during surgery cannot receive naloxone because of the resulting loss of pain relief.

We recently reported that l-cysteine ethyl ester (Mendoza et al., 2013), glutathione ethyl ester (Jenkins et al., 2021), and d-cystine methyl and ethyl esters (Gaston et al., 2021) rapidly reverse the negative effects of opioids, such as morphine and fentanyl, on breathing, Alveolar-arterial gradient (i.e., the index of gas exchange within the lungs) and arterial blood-gas (ABG) chemistry while not reducing opioid analgesia. Since these reduced and oxidized thiol esters block the negative effects of opioids on breathing and because the potent reducing agent N-acetyl-l-cysteine methyl ester does not affect opioid-induced depression of breathing (Baby et al., 2021, it is unlikely that the thiol esters exert their effects by altering the redox state of cells involved in the effects of opioids on breathing. In support of this, there is no real consensus as to whether opioids induce oxidative stress. For example, morphine, buprenorphine and methadone can increase or decrease oxidative stress depending on the experimental conditions (Lee et al., 2004; Almeida et al., 2014; Motaghinejad et al., 2015; Skrabalova et al., 2018; Leventelis et al., 2019).

In another study that we expected to provide evidence that free radicals and superoxide anions play no roles in the ventilatory depressant effects of opioids, we pretreated isoflurane-anesthetized rats with the stable cell permeable free radical scavenger and superoxide dismutase-mimetic agent, Tempol (4-hydroxy-2,2,6,6-tetramethylpiperidine-N-oxyl) (Wilcox and Pearlman, 2008; Wilcox, 2010; Kim et al., 2016), and determined the cardiorespiratory and analgesic effects elicited by subsequent injection of fentanyl (Baby et al., 2021). To our surprise, Tempol markedly blunted the fentanyl-induced decreases in ventilation and arterial blood pressure whereas it did not blunt the antinociceptive actions of fentanyl. It seems somewhat unlikely that the effects of Tempol are only due to alterations in oxidative stress status since unlike Tempol, the powerful antioxidant and superoxide anion scavenger, N-acetyl-l-cysteine methyl ester, did not affect fentanyl-induced suppression of breathing (Baby et al., 2021). The clinical uses for fentanyl and morphine overlap, but there are many instances where one is preferred over the other (Dahan et al., 2010; 2018; Boom et al., 2012). Moreover, there are substantial similarities, but also equally substantial differences, in the abilities of fentanyl and morphine to affect cardiorespiratory parameters in humans and animals (Dahan et al., 2010; 2018; Boom et al., 2012; Henderson et al., 2014; Torralva and Janowsky, 2019) for reasons including, differential actions of the parent opioids and their metabolites on non-opioid receptors (Torralva et al., 2020). Questions arising from our first report on Tempol (Baby et al., 2021) ask 1) whether the presence of isoflurane anesthesia is a factor in the ability of Tempol to blunt the negative cardiorespiratory effects of fentanyl; 2) whether Tempol uniquely affects fentanyl or is active against other opioids; and 3) whether the efficacy of Tempol is related to it being a pretreatment. Obviously, development of therapeutic agents that effectively *reverse* the negative effects of opioids on cardiorespiratory function without directly interacting with opioid receptors would be of great value. Accordingly, we are actively exploring the ability of Tempol and its analogues (Baby et al., 2021) to *reverse* the negative effects of fentanyl and morphine on breathing and cardiovascular parameters in freely-moving rats. The fentanyl studies are on-going, but we have completed the morphine studies and present them here. More specifically, to address some of the above questions and to further explore the potential of Tempol as a therapeutic to effectively treat opioid-induced respiratory depression, we determined whether Tempol could *reverse* the negative effects of *morphine* on ABG chemistry and tissue O_2_ saturation (SpO_2_) in unanesthetized Sprague Dawley rats.

## Materials and Methods

### Ethics Statements

All animal studies were carried out in accordance with the National Institutes of Health Guide for the Care and Use of Laboratory Animals (NIH Publication No. 80.23) revised in 1996. The protocols were approved by the Institutional Animal Care and Use Committee at *Galleon Pharmaceuticals, Inc* (Horsham, PA).

### Animals and Preparations

Adult male Sprague Dawley rats (270–300 g, body weight) with femoral vein and femoral artery catheters in place were obtained from Harlan Laboratories, Inc (Indianapolis, IN). The rats were caged in a Innocage IVC rat caging system (InnoVive, San Diego, CA, United States) with standard housing conditions with free access to food and water. The vivarium temperature (22°C), humidity (35–40%) and light-dark cycle (12:12 h) were maintained consistently. On the day of the experiment, the rats were acclimatized to a 9″ x 5″ plastic container prior to the experiment. After the acclimation period, the venous catheter was extended 15″ with additional micro-renathane tubing (Braintree Scientific, Braintree, MA) with pin connectors attached to the catheter. The arterial catheter was extended 15” with additional micro-renathane tubing with a 3-way connector (Braintree Scientific, MA). One end of the three-way connector was connected to a heparinized saline filled pressure transducer (SP844-28; Memscap Inc. North Carolina, United States of America) to measure arterial blood pressures and heart rate. The other end of the 3-way connector was attached to heparinized saline filled syringe to collect arterial blood samples for arterial blood gases and pH measurement. In these studies described below, the doses of Tempol were chosen on the basis of other rat studies (Xu et al., 2004; Wilcox and Pearlman, 2008; Wilcox, 2010) and from preliminary studies in our laboratory.

### Arterial Blood Gases and pH Measurement

To collect arterial blood samples, any residual saline between the rat and the tubing connecting the syringe was removed and 250 µL of arterial blood was collected with a pre-heparinized 1 ml syringe. The syringe was capped and gently rotated up and down for 3 s to mix the heparin (Hann’s Pharma, Wilmington, DE) and blood to prevent any clotting in the blood gas machine. The sample was immediately injected into the blood gas analyzer, ABL 800 Flex (Radiometer, Westlake, OH) for the determination of pH, pCO_2_, pO_2_, sO_2_ and Alveolar-arterial (A-a) gradient. These samples were taken twice (at time-points 0 and 5 min) before the injection of morphine to ensure patency of the catheters and to obtain reliable baseline blood gases and pH values. Immediately following the second blood collection (at time-point 5 min), all rats received morphine (10 mg/kg, IV) given as a slow bolus in order to induce cardiorespiratory depression. Blood samples were taken 7 and 12 min later (12 and 17 min after initial blood samples were collected). Immediately after the second blood sample was taken (12 min post-morphine), the rats received a slow bolus injection of vehicle or Tempol at 60 or 100 mg/kg IV *via* the venous catheter. Arterial samples were taken 10 and 15 min afterwards (27 and 32 min post-morphine, after initial blood samples were collected, respectively).

### Tissue O_2_ Saturation (SpO_2_) Analysis

SpO_2_ in conscious freely-moving rats was measured continuously using a MouseOx collar sensor (Starr Life Sciences Corp. Oakmont, PA, United States of America) placed over the carotid artery. On the day of the experiment, the rats were allowed to acclimatize to a 9″ x 5” plastic container. The MouseOx collar sensor fitted for the rat neck was connected to STARR-Link, an analog output module in conjunction with the MouseOx plus that converted the calculated parameters to an analog voltage output. The Starr-Link device placed outside the rat cage was interfaced with a PowerLab (ADInstruments, Inc. Colorado, CO, United States of America) and the data were digitized and continuously recorded using LabChart Pro7 software (ADInstruments, CO). SpO_2_ values were averaged in 30 s time bins to calculate the effects of Tempol on morphine-induced respiratory depression. More specifically, all of the rats received a slow bolus injection of morphine (10 mg/kg, IV) and after 15 min, the rats received a slow bolus injection of vehicle or Tempol at 60 or 100 mg/kg, IV. SpO_2_ was monitored for an additional 15 min.

### Blood Pressure and Heart Rate Measurement

Blood pressure parameters and heart rate were continuously recorded in unrestrained freely-moving rats directly using femoral intra-arterial catheter as detailed previously (Baby et al., 2021). In brief, the arterial catheter was connected to a heparinized saline-filled pressure transducer (SP844-28; Memscap Inc. North Carolina, United States of America) and blood pressure signals were amplified using the Bridge Amp (FE221, AD Instruments, Inc.). The Bridge Amp was calibrated (2-point calibrations) before each experiment using sphygmomanometer. The arterial blood pressure wave-forms were sampled at 1-2k/sec and band-pass filtered between 0 and 1,000 Hz. Cyclic measurement algorithms in the LabChart 7 pro software (AD Instruments, Inc.) were used to calculate heart rate, diastolic blood pressure (DBP), systolic blood pressure (SBP) and mean arterial blood pressure (MAP) from the arterial blood pressure waveforms. The blood pressure waveforms were digitized (PowerLab, AD Instruments Inc.) and continuously recorded. The blood pressure and heart rate values were averaged every 30 s to calculate the effects of morphine and the Tempol effects on morphine-induced cardiovascular responses. The ratios of heart rate/MAP were determined throughout the experiments to provide an index of baroreceptor heart reflex activity as described previously (Lewis et al., 1999; Salman et al., 2020). With respect to the protocol, all rats received a slow bolus injection of morphine (10 mg/kg, IV) and after 15 min, the rats received a slow bolus injection of vehicle or Tempol at 60 or 100 mg/kg, IV. The cardiovascular parameters were all monitored for a further 15 min.

### Drugs

Saline (vehicle) and morphine sulfate solution (50 mg/ml) were purchased from Hospira Inc (Lake Forest, IL, United States). Working dilutions of morphine (10 mg/ml) was prepared in sterile saline. Tempol was purchased from Tocris Bioscience (Minneapolis, MN, United States) and the stock solution (300 mg/ml) was prepared in sterile saline. Working dilutions (60 mg/ml and 100 mg/ml) were also prepared in sterile saline.

### Data Analyses

All data are presented as mean ± SEM and were evaluated using one-way and two-way ANOVA followed by Bonferroni corrections for multiple comparisons between means using the error mean square term from the ANOVA (Wallenstein et al., 1980). Differences between means were taken to be significant at *p* < 0.05. Statistical analyses were performed using GraphPad Prism software (GraphPad Software, Inc. La Jolla, CA).

## Results

### Effects of Tempol on Morphine-Induced Changes in ABG Chemistry and A-A Gradient

**FIGURE 1:**
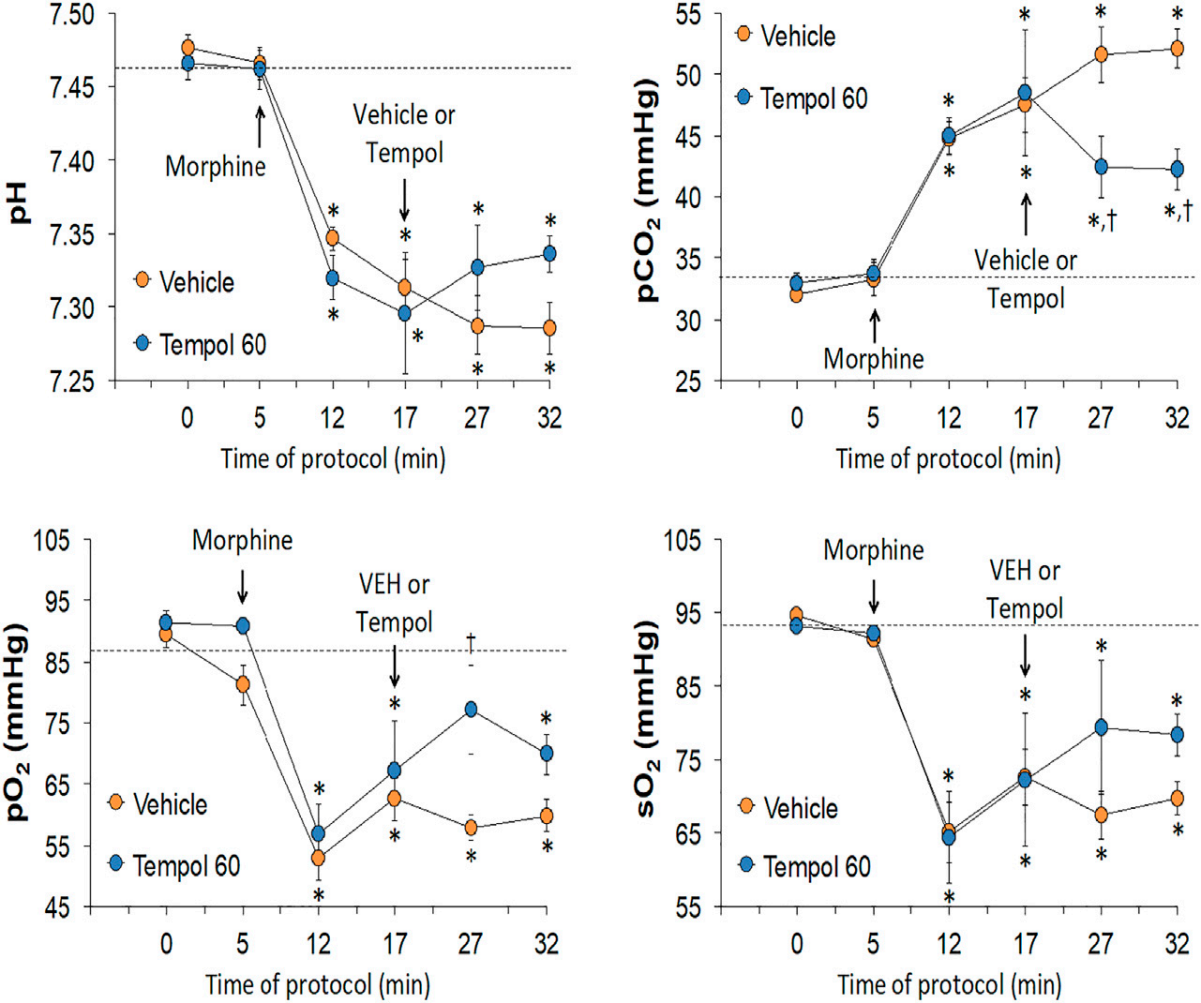
Changes in pH, pCO_2_, pO_2_ and sO_2_ elicited by a bolus injection of morphine (10 mg/kg, IV) and subsequent injection of vehicle (saline) or Tempol (, 60 mg/kg, IV; Tempol 60) in freely-moving rats. The data are presented as mean ± SEM. The numbers of rats in the vehicle and Tempol 60 groups were 7 and 3, respectively. *p < 0.05, significant change from baseline (time 0 value). ^†^ *p* < 0.05, Tempol-induced response *versus* vehicle-induced responses.

**FIGURE 2:**
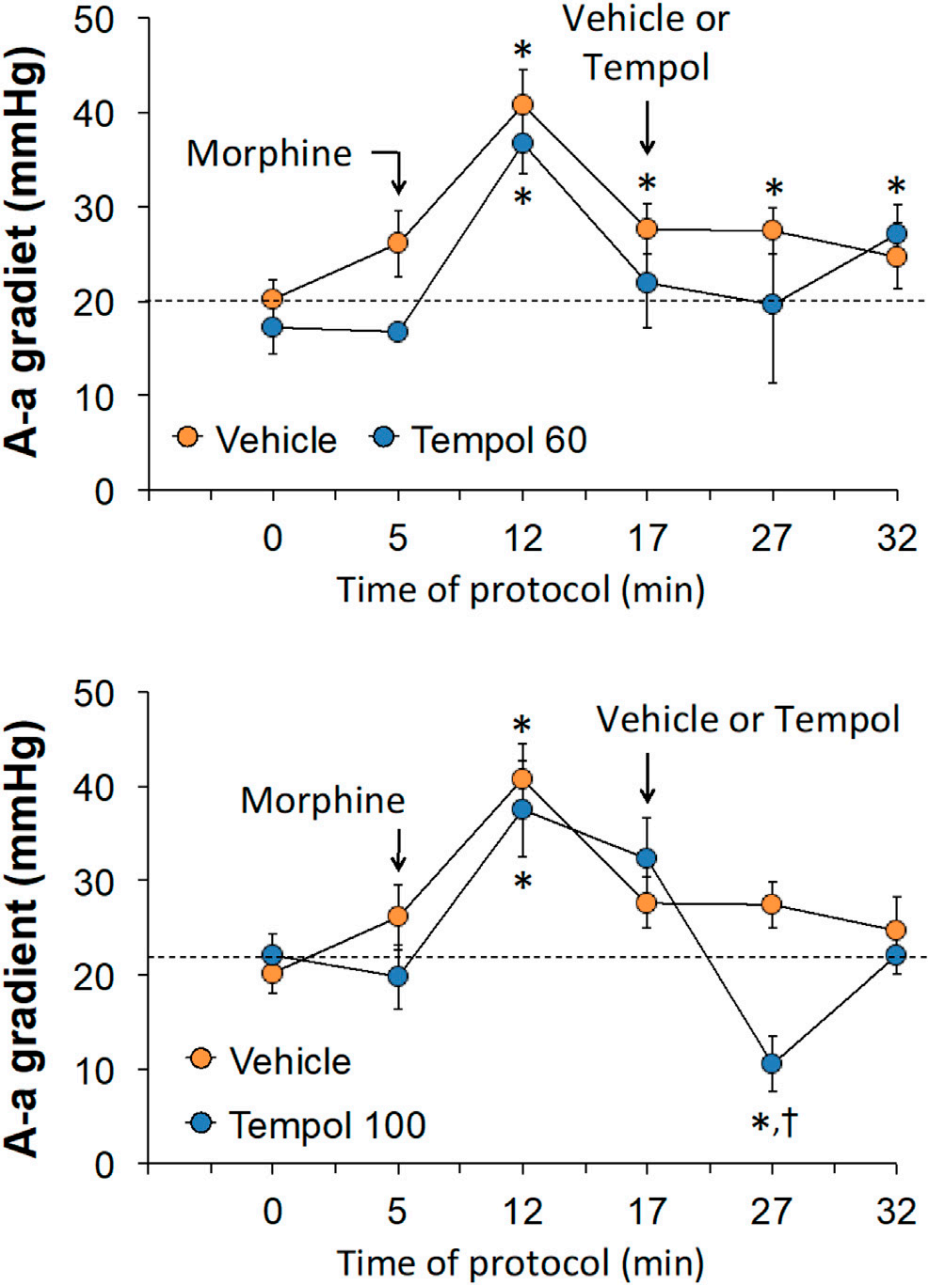
Changes in Alveolar-arterial (A-a) gradient elicited by the bolus injection of morphine (10 mg/kg, IV) and subsequent injection of vehicle (saline) or Tempol at doses of 60 mg/kg, IV (Tempol 60) (top panel) or 100 mg/kg, IV (Tempol 100) (bottom panel) in freely-moving rats. The data are presented as mean ± SEM. The numbers of rats in vehicle, Tempol 60 and Tempol 100 groups were 7, 3 and 7, respectively. *p < 0.05, significant change from baseline (time 0 value). ^†^ *p* < 0.05, Tempol-induced response *versus* vehicle-induced responses.

**FIGURE 3:**
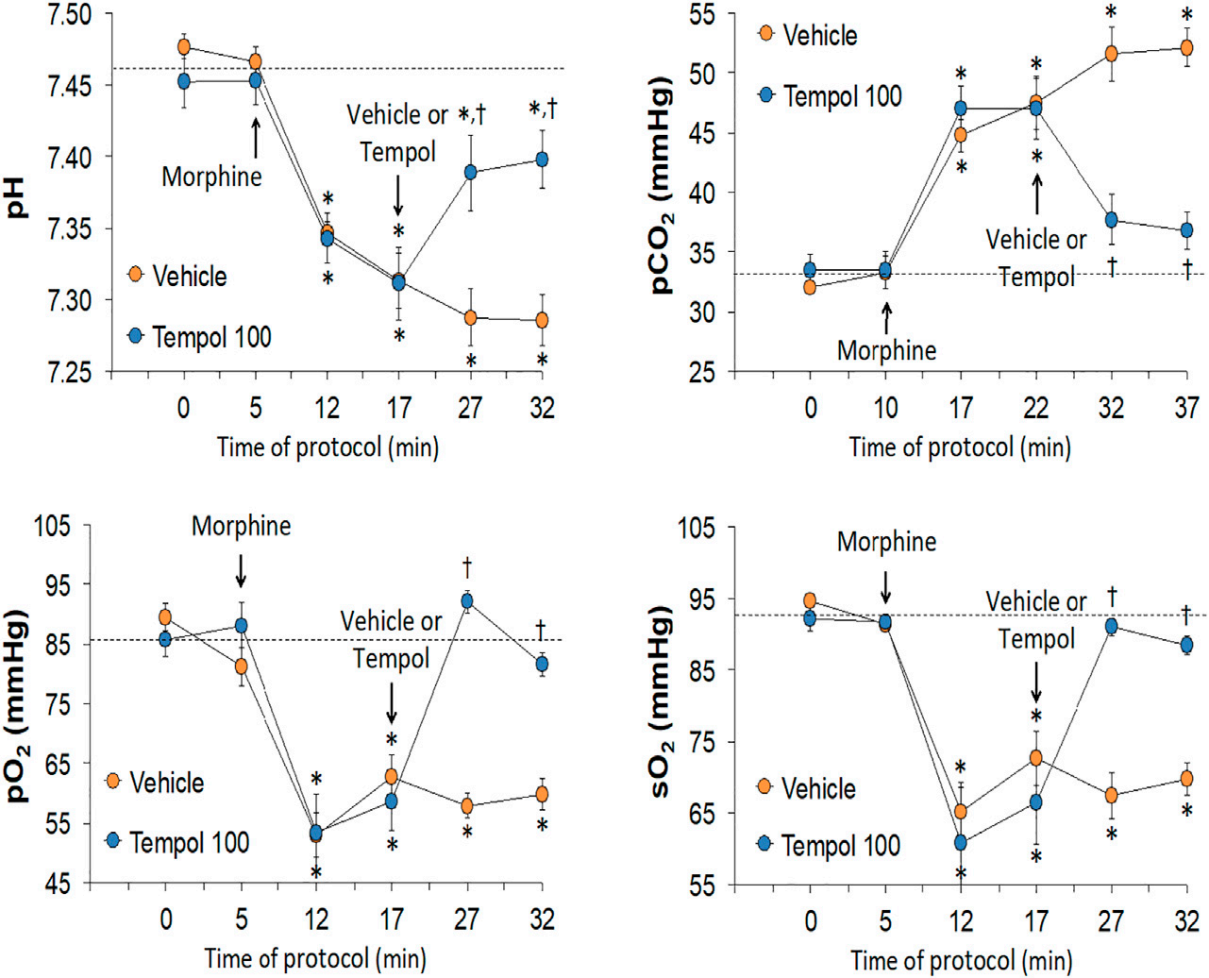
Changes in pH, pCO_2_, pO_2_ and sO_2_ elicited by the bolus injection of morphine (10 mg/kg, IV) and the subsequent injection of vehicle (saline) or Tempol (100 mg/kg, IV; Tempol 100) in freely-moving rats. The data are presented as mean ± SEM. The numbers of rats in the vehicle and Tempol 100 groups were 7 and 7, respectively. *p < 0.05, significant change from baseline (time 0 value). ^†^ *p* < 0.05, Tempol-induced response *versus* vehicle-induced responses.

The changes in pH, pCO_2_, pO_2_ and sO_2_ elicited by the bolus injection of morphine (10 mg/kg, IV) and the subsequent injection of vehicle (saline) or a 60 mg/kg dose of Tempol (Tempol 60) in freely-moving rats are summarized in Figure 1. The injection of morphine elicited pronounced decreases in pH, pO_2_ and sO_2_ that were accompanied by equally pronounced increases in pCO_2_. These responses were similar in magnitude in the two groups. The injection of vehicle did not alter the morphine-induced responses whereas the 60 mg/kg dose of Tempol reversed the negative effects of morphine on pCO_2_ and pO_2_ with the effects on pH and sO_2_ not reaching significance. As seen in the top panel of Figure 2, the changes in pCO_2_ and pO_2_ elicited by morphine resulted in a rise in the A-a gradient (indicative of a mismatch of ventilation-perfusion) that was not affected by Tempol at doses 60 or 100 mg/kg. Changes in pH, pCO_2_, pO_2_ and sO_2_ elicited by the injection of morphine (10 mg/kg, IV) and subsequent injection of vehicle (saline) or 100 mg/kg dose of Tempol (Tempol 100) are summarized in Figure 3. The 100 mg/kg dose of Tempol elicited a sustained reversal of the negative effects of morphine on pH, pCO_2_, pO_2_ and sO_2_. Additionally it is important to note that in the bottom panel of Figure 2, the 100 mg/kg dose of Tempol elicited a significant decrease in the A-a gradient 5 min post-administration, whereas at 10 min post-administration there was no difference between the vehicle or Tempol-treated rats.

### Effects of Tempol on Morphine-Induced Changes in SpO_2_

**FIGURE 4:**
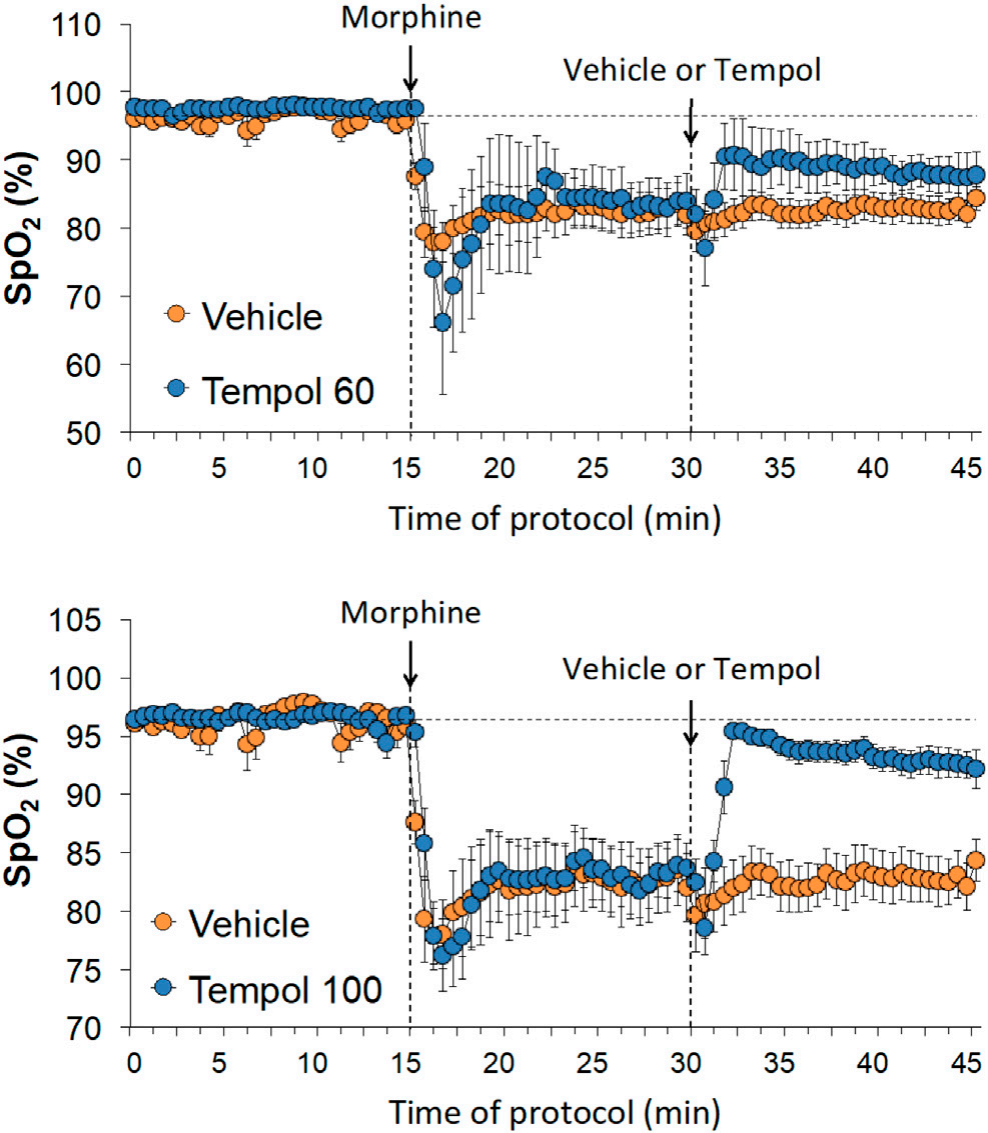
Changes in tissue oxygen saturation (SpO_2_) elicited by the bolus injection of morphine (10 mg/kg, IV) and the subsequent injection of vehicle (saline), Tempol at 60 mg/kg, IV (Tempol 60) or Tempol at 100 mg/kg, IV (Tempol 100) in freely-moving rats. The data are presented as mean ± SEM. The numbers of rats in the vehicle, Tempol 60 and Tempol 100 groups were 9, 3 and 10, respectively.

Baseline SpO_2_ values in the three groups are summarized in Supplemental Table S1. There were no between group differences in baseline SpO_2_ values. The changes in SpO_*2*_ elicited by an injection of morphine (10 mg/kg, IV) and subsequent injection of vehicle (saline), a 60 mg/kg dose of Tempol (Tempol 60) or a 100 mg/kg dose of Tempol (Tempol 100) in freely-moving rats are summarized in Figure 4. Morphine elicited a pronounced and long-lasting decrease in SpO_2_ that was not obviously affected by injection of vehicle. The injection of the 60 mg/kg dose of Tempol (Tempol 60) elicited a minor short-lived rise in SpO_2_, whereas the 100 mg/kg dose of Tempol (Tempol 100) elicited a prompt and sustained reversal of the negative effects of morphine on tissue oxygenation. As summarized in Table 1, the injection of morphine elicited similar cumulative decreases in SpO_2_ in the three groups (sum of responses that occurred from 0–15 min after injection). The injection of vehicle did not change the effects of morphine, such that the cumulative decrease in SpO2 over the 0–15 min post-vehicle injection phase (−14.2 ± 2.2) was similar to the 0–15 min post-morphine injection phase (−14.5 ± 3.4). In addition, the cumulative %change that occurred 0–30 min after injection of vehicle (using the 15 min post-morphine value as the pre-value) was not significant. In contrast, the cumulative responses after injection of the 60 mg/kg (Tempol 60) and 100 mg/kg (Tempol 100) doses of Tempol were significant with the 100 mg/kg dose being clearly stronger.

**TABLE 1:** Cumulative changes in SpO_2_ values elicited by morphine and Tempol.

phase	Vehicle	Tempol 60	Tempol 100
Morphine, %change from baseline	−14.5 ± 3.4*	−15.2 ± 3.5*	−14.9 ± 2.5*
Post-drug (0–15 min), %change from baseline	−14.2 ± 2.2*	−9.6 ± 4.0*	−4.5 ± 0.6*^,†^
Post-drug (0–30 min), %change from morphine	+0.9 ± 1.2	+5.0 ± 0.6*^,†^	+12.4 ± 2.0*^,†^

Data are presented as mean ± SEM. The dose of morphine was 10 mg/kg, IV. The terms Tempol 60 and Tempol 100 refer to IV doses of 60 mg/kg and 100 mg/kg, respectively. The numbers of rats in the vehicle, Tempol 60 and Tempol 100 groups were 9, 3 and 10, respectively. *p < 0.05, significant response. ^†^ *p* < 0.05, Tempol-induced response *versus* vehicle-induced responses.

### Effects of Tempol on Morphine-Induced Changes in Cardiovascular Parameters

**FIGURE 5:**
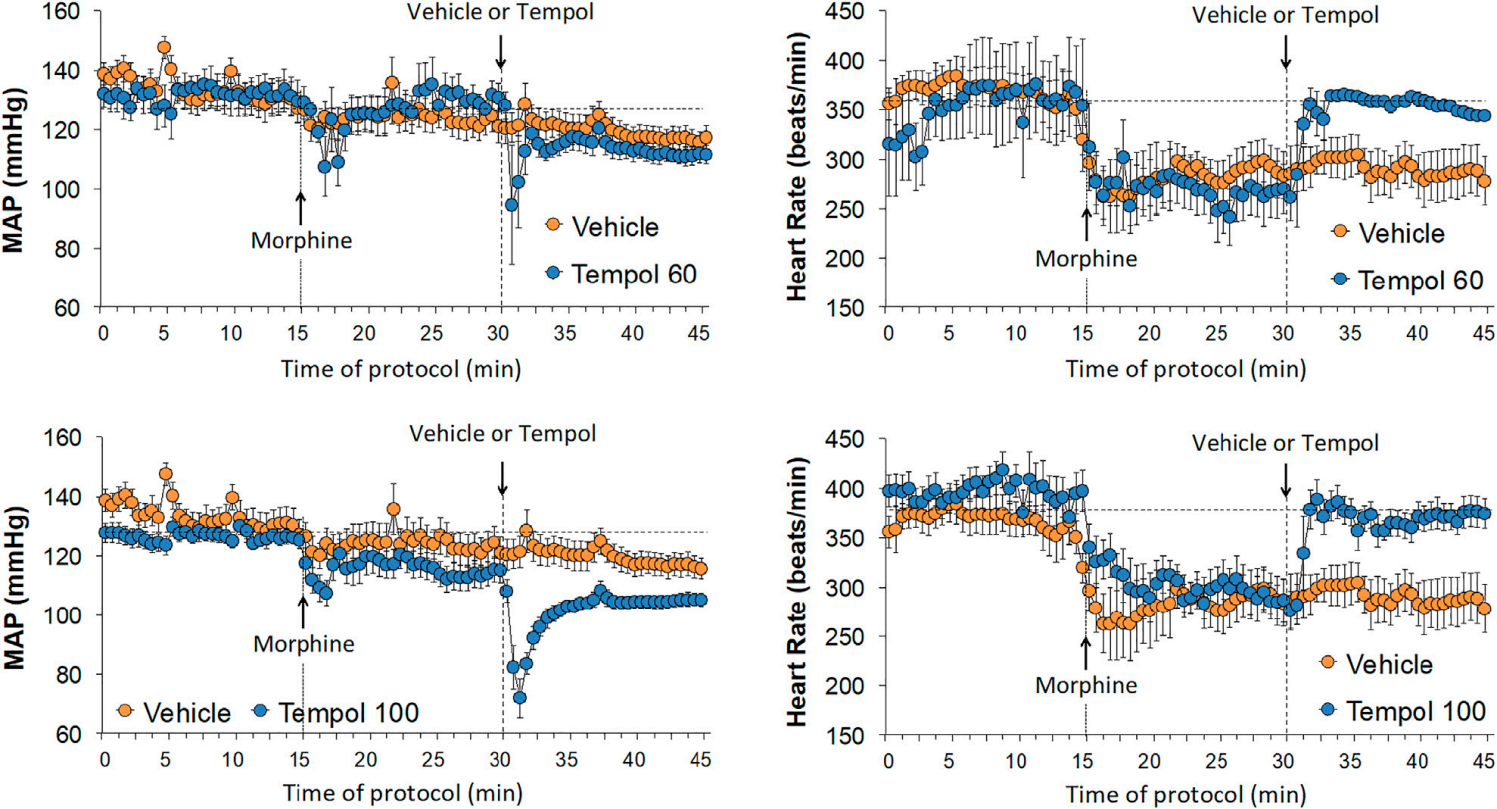
Changes in mean arterial blood pressure (MAP) and heart rate elicited by bolus injection of morphine (10 mg/kg, IV) and the subsequent injection of vehicle (saline), Tempol at 60 mg/kg, IV (Tempol 60) or Tempol at 100 mg/kg, IV (Tempol 100) in freely-moving rats. Data are shown as mean ± SEM. The numbers of rats in vehicle, Tempol 60 and Tempol 100 groups were 9, 3 and 10, respectively.

Baseline cardiovascular values in the three groups are summarized in Supplemental Table S1. There were no between group differences in any of the parameters. The changes in mean arterial blood pressure (MAP) and heart rate elicited by the injection of morphine (10 mg/kg, IV) and subsequent injection of vehicle (saline), a 60 mg/kg dose of Tempol (Tempol 60) or a 100 mg/kg dose of Tempol (Tempol 100) in freely-moving rats are summarized in Figure 5. Morphine elicited relatively minor initial decreases MAP but substantial decreases in heart rate. The injection of the 60 mg/kg dose of Tempol (Tempol 60) elicited a minor short-lived fall in MAP whereas the 100 mg/kg dose of Tempol (Tempol 100) elicited a prompt and sustained decrease in MAP. The 60 and 100 mg/kg doses of Tempol elicited a prompt and sustained reversal of the morphine-induced bradycardia. The changes in diastolic (DBP) and systolic (SBP) arterial blood pressures are summarized in Supplemental Figure S1. The data shows that changes in both DBP and SBP contribute equally to those described for MAP (see above). Moreover, as can be seen in Supplemental Figure S2, the changes in MAP and heart rate result in somewhat minor reductions in the ratio of heart rate/MAP (i.e., the index of baroreceptor heart rate reflex activity) following injection of morphine, suggesting a loss of baroreflex activity. The injection of Tempol, specifically at 100 mg/kg, elicited rapid and sustained increase in the heart rate/MAP ratio suggestive of pronounced increases in baroreceptor heart rate reflex activity. As summarized in Table 2, the injection of morphine elicited relatively minor cumulative decreases (about 10% or less) in DBP, SBP and MAP in the three groups of rats (sum of responses that occurred from 0–15 min post-injection). The cumulative decreases in DBP, SBP and MAP over the 0–15 min post-vehicle injection phase were similar to, or significantly greater than, those recorded during the 0–15 min post-morphine injection phase. In addition, the cumulative %change that occurred 0–15 min after injection of vehicle (using the final 15 min morphine value as the pre-value) were not significant for SBP or MAP. In contrast, the cumulative responses in DBP, SBP and MAP following the injection of the 60 and the 100 mg/kg doses of Tempol (as recorded between 15 and 30 min post-morphine) were all greater than those recorded between 0 and 15 min post-morphine injection. As also seen in Table 2, morphine elicited significant and similar cumulative decreases in heart rate and heart rate/MAP in the three groups of rats and that both doses of Tempol elicited sustained reversal of these effects of morphine.

**TABLE 2:** Cumulative changes in MAP values elicited by morphine and Tempol.

Parameter	Phase	Vehicle	Tempol (mg/kg, IV)	
			Tempol 60	Tempol 100
DBP, mmHg	Morphine, %change from baseline	−10.5 ± 4.3*	−7.3 ± 3.0	−10.3 ± 3.5*
	Post-drug, %change from baseline	−12.9 ± 4.1*	−16.7 ± 1.7*	−19.6 ± 2.0*
	Post-drug, %change from morphine	−3.8 ± 0.1*	−13.9 ± 2.4*^,†^	−3.2 ± 4.1*
SBP, mmHg	Morphine, %change from baseline	−3.6 ± 2.8	0.2 ± 2.8	−4.6 ± 2.5*
	Post-drug, %change from baseline	−7.5 ± 3.1*	−9.5 ± 1.9*	−18.8 ± 1.2*^,†^
	Post-drug, %change from morphine	−1.0 ± 1.6	−11.3 ± 1.6*^,†^	−9.1 ± 2.3*^,†^
MAP, mmHg	Morphine, %change from baseline	−7.5 ± 3.9*	−3.6 ± 3.8	−8.2 ± 3.1*
	Post-drug, %change from baseline	−10.6 ± 3.7*	−13.7 ± 1.7*	−19.4 ± 1.5*^,†^
	Post-drug, %change from morphine	−0.6 ± 1.4	−13.2 ± 1.8*^,†^	−5.6 ± 3.2*
Heart rate (HR), beats/min	Morphine, %change from baseline	−23.9 ± 5.9	−22.2 ± 3.2*	−23.7 ± 2.9*
	Post-drug, %change from baseline	−20.7 ± 5.3	+1.8 ± 7.1^†^	−6.8 ± 3.0*^,†^
	Post-drug, %change from morphine	+3.4 ± 5.6	+31.2 ± 10.3*^,†^	+37.0 ± 9.9*^,†^
HR/MAP, mmHg/beats/min	Morphine, %change from baseline	−14.2 ± 6.1*	−19.2 ± 2.5*	−14.3 ± 3.5*
	Post-drug, %change from baseline	−9.0 ± 5.3	+19.2 ± 12.7^†^	+18.3 ± 3.9*^,†^
	Post-drug, %change from morphine	+7.4 ± 5.6	+53.3 ± 13.7*^,†^	+43.0 ± 7.1*^,†^

Data are presented as mean ± SEM. The dose of morphine was 10 mg/kg, IV. The terms Tempol 60 and Tempol 100 refer to IV doses of 60 mg/kg and 100 mg/kg, respectively. The numbers of rats in the vehicle, Tempol 60 and Tempol 100 groups were 9, 3 and 10, respectively. *p < 0.05, significant response. ^†^ *p*< 0.05, Tempol-induced response *versus* vehicle-induced responses.

## Discussion

The present study confirms that morphine (10 mg/kg, IV) has pronounced deleterious effects on ABG chemistry (decreases in pH, pO_2_ and sO_2_ accompanied by increases in pCO_2_) and noticeable decreases in gas-exchange in the lungs (i.e., elevated A-a gradient) in freely-moving male Sprague Dawley rats (May et al., 2013; Gaston et al., 2021). This study also confirms the findings of Grinnell et al. (2014) that morphine elicits a substantial decrease in tissue oxygenation saturation (SpO_2_) in rats. The administration of the 10 mg/kg dose of morphine elicited relatively minor reductions in DBP, SBP and MAP in our freely-moving rats with the available literature reporting minimal to substantial reductions in these parameters in non-anesthetized rats (Della Puppa et al., 1989; Thornhill et al., 1989; Thurston et al., 1993). Consistent with published literature, morphine also elicited robust and sustained decreases in heart rate, which have been reported to mainly involve an increase in vagal drive to the heart (Della Puppa et al., 1989; Thornhill et al., 1989; Thurston et al., 1993). The ratio of heart rate/MAP can be taken as an index of baroreceptor reflex activity (Lewis et al., 1999; Salman et al., 2020), and our data provide evidence that morphine decreased this ratio and therefore potentially decreased the activity of the baroreflex in our rats. There is remarkably little published data as to the exact effects of morphine or any other opioid on the baroreceptor reflex system in freely-moving (non-anaesthetized) rats although the reported evidence we could find demonstrated that morphine enhances baroreceptor reflex activity in anesthetized rats (Shanazari et al., 2011) whereas it inhibits baroreflex activity in humans (Kotrly et al., 1984).

A major finding of the present study was that the intravenous injection of Tempol and especially at a 100 mg/kg dose was able to immediately reverse the pronounced negative effects of a 10 mg/kg intravenous dose of morphine on ABG chemistry, A-a gradient and SpO_2_ in the freely-moving rats. These findings complement our earlier report that pretreatment with a 100 mg/kg dose of Tempol markedly blunted the deleterious changes in ABG chemistry, A-a gradient and ventilatory parameters (e.g., frequency of breathing, tidal volume and minute ventilation) elicited by the subsequent injection of fentanyl in isoflurane-anesthetized rats (Baby et al., 2021). The present study did not directly address the mechanisms underlying the ability of Tempol to reverse the negative effects of morphine on ventilatory control processes pertinent to our study, however, the rapid and sustained effects of Tempol on ABG chemistry and SpO_2_ along with the relatively transient effects on A-a gradient (improved gas exchange in the lungs of morphine-treated rats) certainly suggests that the primary reason for the improved status of the ABG chemistry is due to the ability of Tempol to increase ventilatory performance (e.g., enhanced minute ventilation and inspiratory drive) in the morphine-treated rats.

The present study also found that the 60 and 100 mg/kg doses of Tempol elicited pronounced and sustained decreases in MAP in morphine-treated rats that were similar in magnitude to those that occur in naïve rats (Baby et al., 2021). The reductions in MAP in naïve rats is due to the direct *activation* of Ca^2+^-activated K^+^-channels (BK_Ca_) channels in vascular smooth muscle rather than by scavenging free radicals and/or superoxide anion (Xu et al., 2004; 2005; 2006). The systemic administration of Tempol produces mild to profound dose-dependent bradycardia in freely-moving rats (Xu et al., 2004; 2005; 2006). Rather remarkably, the injection of Tempol (either 60 or 100 mg/kg) caused a pronounced and sustained increase in heart rate by mechanisms that we are currently exploring. We can only conjecture that Tempol somehow inhibits the signaling central signaling pathways by which morphine diminishes heart rate, including the activation of vagal drive (Della Puppa et al., 1989; Thornhill et al., 1989; Thurston et al., 1993). It is also important to consider the possibility that the increase in ventilation and improvements in gas-exchange within the lungs and therefore ABG chemistry following Tempol administration in morphine-treated rats, may be a result of the large reduction in blood pressure elicited by Tempol. For example, Viires et al. (1983) reported that the hypotension induced by pericardial temponade elicited an increase in ventilation in dogs by an increase in blood flow to the diaphragm (via a redistribution of systemic blood flow).

Currently, we do not have any knowledge about or any understanding of the molecular mechanisms by which Tempol reverses the negative effects of morphine on ABG chemistry and SpO_2_. However, we do believe that the ability of Tempol to activate BK_Ca_ channels (Xu et al., 2005; 2006) is most likely not a key contributor to the mechanisms by which Tempol reverses morphine-induced depression of ventilatory parameters responsible for the observed robust changes in ABG chemistry and SpO_2_ in the morphine-treated rats. Primary reasons for this belief are that 1) the activation of BK_Ca_ channels substantially diminishes carotid body glomus cell activity (Pichard et al., 2015; Wang et al., 2018) thereby dampening breathing, and 2) because pharmacological blockade of BK_Ca_ channels partially reverses opioid-induced respiratory depression in humans and animals (McLeod et al., 2014; Roozekrans et al., 2014; Dallas et al., 2015; Golder et al., 2015). Although the mechanisms responsible for the cardiovascular effects of morphine are multi-factorial, the direct and endothelium-dependent vasodilation of systemic and pulmonary arteries are both important mechanisms contributing to the underlying morphine-induced hypotension and the distribution of blood flow within the pulmonary circulation, respectively (Feuerstein, 1985; Johnson et al., 1985; Kaye et al., 2008; Toda et al., 2009; Behzadi et al., 2018). Nonetheless, though direct evidence is lacking, it would be reasonable to suggest that the ability of Tempol to reverse the negative effects of morphine on MAP involves effects on central (e.g., brainstem) circuitry and peripheral (e.g., sympathetic and parasympathetic ganglia and nerve terminals) that directly participate in the expression of the hypotension elicited by morphine or those that modulate this response (Feuerstein, 1985; Johnson et al., 1985; Toda et al., 2009; Behzadi et al., 2018). We have not determined whether the actions of Tempol seen in this study involve its proven capacity to scavenge free radicals and superoxide anion. However, our findings with Tempol against fentanyl (Baby et al., 2021) and morphine (present study) raise the possibility that Tempol, which is clinically approved to treat alopecia in humans (Wilcox and Pearlman, 2008; Wilcox, 2010), and many commercially-available Tempol analogues (Baby et al., 2021; Supplemental Figure S9) may be repurposed as an intravenous agent to both prevent and reverse the profound negative effects of fentanyl and morphine on breathing and arterial blood pressure while preserving analgesia in human subjects.

In summary, the present study extends our findings regarding the beneficial effects of Tempol against the negative effects of fentanyl on breathing, ABG chemistry and gas-exchange in the lungs, and the potential problems regarding the ability of Tempol to reduce arterial blood pressure (Baby et al., 2021). Previous studies have demonstrated the beneficial effects of Tempol in cell and animal models of neurodegenerative diseases, shock, hypertension, diabetes, ischemia-reperfusion injury, traumatic brain injury, tumororigenesis, chemotherapy-induced neuropathic pain and alopecia (Wilcox and Pearlman, 2008; Wilcox, 2010; Kim et al., 2016; Bernardy et al., 2017; Wang et al., 2018; Afjal et al., 2019; Chiarotto et al., 2019). Future studies with the array of commercially available Tempol analogues (Baby et al., 2021; Supplemental Figure S9) will help to define structure-activity relationships with respect to the wanted and unwanted effects of Tempol-like drugs. The findings may pave the way for the development of a novel series of drugs that can be used to overcome opioid-induced respiratory and hemodynamic depression. Of course, it is possible that designing Tempol analogues that do not have the hypotensive and vasodilator activities of Tempol may ultimately also eliminate efficacy against the negative effects of opioids on ventilatory function.

### Study Limitations

The present study has several limitations that will be subject to future research studies in which we will 1) administer Tempol following the injection of fentanyl or even higher potency analogues (e.g., sufentanil) in freely-moving male and female rats, in order to determine the efficacy of Tempol as a reversal agent against the profound cardiorespiratory responses that are elicited by these high potency synthetic opioids, and 2) inject Tempol following injection of fentanyl (following bolus or infusion paradigms) in combination with diazepam and/or methamphetamine in freely-moving male and female rats to provide information as to the efficacy of Tempol against the cardiorespiratory effects of these combinations, which are ever increasing real-world scenarios (Warner et al., 2016; Macleod et al., 2019; Baker et al., 2021). Another important limitation of this study was that end-tidal PO_2_ and PCO_2_ were not continuously monitored. Such measurements would allow us to determine the temporal relationships between the changes in alveolar gases, ventilatory parameters, and arterial blood pressures because of morphine administration and subsequent administration of Tempol.

Moreover, this study only investigated male rats. Future studies must incorporate female animals to better understand the efficacy profile of Tempol. Additionally in our Tempol studies we have not established any of the molecular mechanisms by which readily cell-penetrant Tempol prevents and reverses the negative effects of fentanyl (Baby et al., 2021) and morphine (present study) on cardiorespiratory function, although published data suggests that it is unlikely to involve modulation of BK_Ca_ channels (Baby et al., 2021), or direct blockade of opioid receptors on the basis that Tempol spares morphine analgesia (Baby et al., 2021). With respect to potential plasma membrane and intracellular targets for Tempol, our collaborator Dr. Christopher Ellis (DEVCOM Chemical Biological Center, US Army) is employing phase (*Public Health Assessment via Structural Evaluation*) technology (Ellis et al., 2019) and molecular docking methods (Ellis et al., 2018) to identify protein sites (e.g., G-protein-coupled receptors, ion-channels, and membrane and intracellular enzymes) to which Tempol, morphine and fentanyl bind to, with emphasis on the signaling proteins/pathways most relevant to opioid-induced cardiorespiratory depression (Ellis et al., 2018). As mentioned previously (Baby et al., 2021), we will initially develop binding profiles for each drug of interest (e.g., Tempol, fentanyl) and then use phase to identify functionally relevant protein targets to develop potential insights into the mechanisms of action of Tempol. These studies will be augmented by molecular docking modeling approaches to predict binding affinities and displacement coefficients of the drugs at the sites identified in the first stage (phase computations) of these studies. This information from Dr. Ellis will play a vital role in our understanding of the functional interactions between Tempol and opioids with respect to protein targets and will directly inform future studies designed to optimize Tempol and derivatives to treat the negative effects of opioids while maintaining beneficial effects, such as analgesia.

## Conclusion

The findings that Tempol reverses the negative effects of morphine on ABG chemistry and SpO_2_ in freely-moving Sprague Dawley rats is an important addition to the development of drugs to combat opioid-induced depression of respiratory and cardiovascular systems. From this and our earlier study (Baby et al., 2021) we now know that 1) Tempol is efficacious as a pretreatment against fentanyl (Baby et al., 2021), 2) Tempol is a reversal agent against morphine (present study), 3) Tempol is effective in isoflurane-anesthetized rats (Baby et al., 2021) and in freely-moving rats (present study), and 4) Tempol is efficacious against both fentanyl and morphine. Reversal strategies with a drug with the pharmacological profile of Tempol can be used in many clinical scenarios, such as in the operating room when the situation requires immediate and sustained reversal of opioid-induced respiratory depression without compromising analgesia, and in the increasingly greater set of scenarios when unintended/intended overdoses with an opioid occur. Future work involving elucidating the effects of Tempol on the intracellular signaling pathways activated by morphine and fentanyl, will entail examination of whether Tempol directly interacts with signaling pathways or modifies there activities indirectly by scavenging free radicals and/or superoxide anion. We will compare the efficacy of Tempol to free radical/superoxide anion scavengers with differing chemical structures using isothermal titration calorimetry on proteins within the morphine/fentanyl signaling pathway, high throughput screening methods including surface plasmon resonance and hydrogen deuterium exchange mass spectrometry (Marcsisin and Engen, 2010; Nguyen et al., 2015; Baranauskiene et al., 2019). These future studies are intended to define the precise signaling mechanisms by which Tempol prevents and reverses fentanyl- or morphine-induced depression of ventilatory function and arterial blood pressure without affecting analgesia/antinociception.

## Data Availability

The raw data supporting the conclusions of this article will be made available by the authors, without undue reservation.

## Abbreviations

A-a gradient, Alveolar-arterial gradient; ABG, arterial blood-gas chemistry; BKCa, Ca2+-activated K+-channels; DBP, diastolic arterial blood pressure; Tempol, 4-hydroxy-2,2,6,6-tetramethylpiperidine-N-oxyl; MAP, mean arterial blood pressure; SBP, systolic arterial blood pressure.
