# Supplementary material for: Tempol Reverses the Negative Effects of Morphine on Arterial Blood-Gas Chemistry and Tissue Oxygen Saturation in Freely-Moving Rats
Source: Front Pharmacol. 2021 Sep 22;12:749084. doi: 10.3389/fphar.2021.749084 (PMC8493249; doi:10.3389/fphar.2021.749084)
Supplement: Supplementary file 1 [file DataSheet1.pdf]

Supplemental File

Tempol reverses the negative effects of morphine on arterial blood-gas chemistry and tissue oxygen saturation in freely-moving rats

Santhosh M. Baby,[1,†] Joseph F. Discala,[1] Ryan Gruber,[1] Paulina M. Getsy,[2] Feixiong Cheng,[3] Derek S. Damron,[4] Stephen J. Lewis[2,5]*

[1]Galleon Pharmaceuticals, Inc., 213 Witmer Road, Horsham, PA, USA

[2]Department of Pediatrics, Case Western Reserve University, Cleveland, OH, USA

[3]Genomic Medicine Institute, Lerner Research Institute, Cleveland Clinic, Cleveland, OH, USA

[4]Department of Biological Sciences and School of Biomedical Sciences, Kent State University, Kent, OH, USA

[5]Department of Pharmacology, Case Western Reserve University, Cleveland, OH, USA

†Current address: Santhosh M. Baby, Translational Sciences Treatment Discovery, Galvani Bioelectronics, Inc., 1250 S Collegeville Rd., Collegeville, Pennsylvania 19426.
Email: santhosh.m.baby@galvani.bio

***Corresponding Author:**
Stephen J. Lewis, PhD
Department of Pediatrics, Division of Pulmonology, Allergy and Immunology
Department of Pharmacology School of Medicine
Biomedical Research Building, Room 831
Case Western Reserve University
10900 Euclid Avenue
Cleveland, OH 44106-4984
Phone: 216-368-3482
Email: sjl78@case.edu

Supplemental Table 1. Baseline parameters

		Tempol (mg/kg, IV)	
**Parameter**	**Saline (ml/kg)**	**60**	**100**
Number of rats	9	3	10
SpO_2, %	96.0 ± 0.5	97.5 ± 0.3	96.6 ± 0.5
SBP, mmHg	129 ± 5	129 ± 5	120 ± 2
DBP, mmHg	148 ± 4	139 ± 5	136 ± 3
MAP, mmHg	135 ± 4	132 ± 5	126 ± 3
Heart rate, beats/min	368 ± 13	352 ± 43	393 ± 15
Heart rate/MAP, mmHg/beats/min	2.9 ± 0.2	2.7 ± 0.4	3.1 ± 0.1

SBP, systolic arterial blood pressure. DBP, diastolic arterial blood pressure. MAP, mean arterial blood pressure. SpO_2, tissue O_2 saturation. The data are presented as mean ± SEM. There were no between group differences for any parameter ($P > 0.05$ for all comparisons).

Supplemental Figure 1

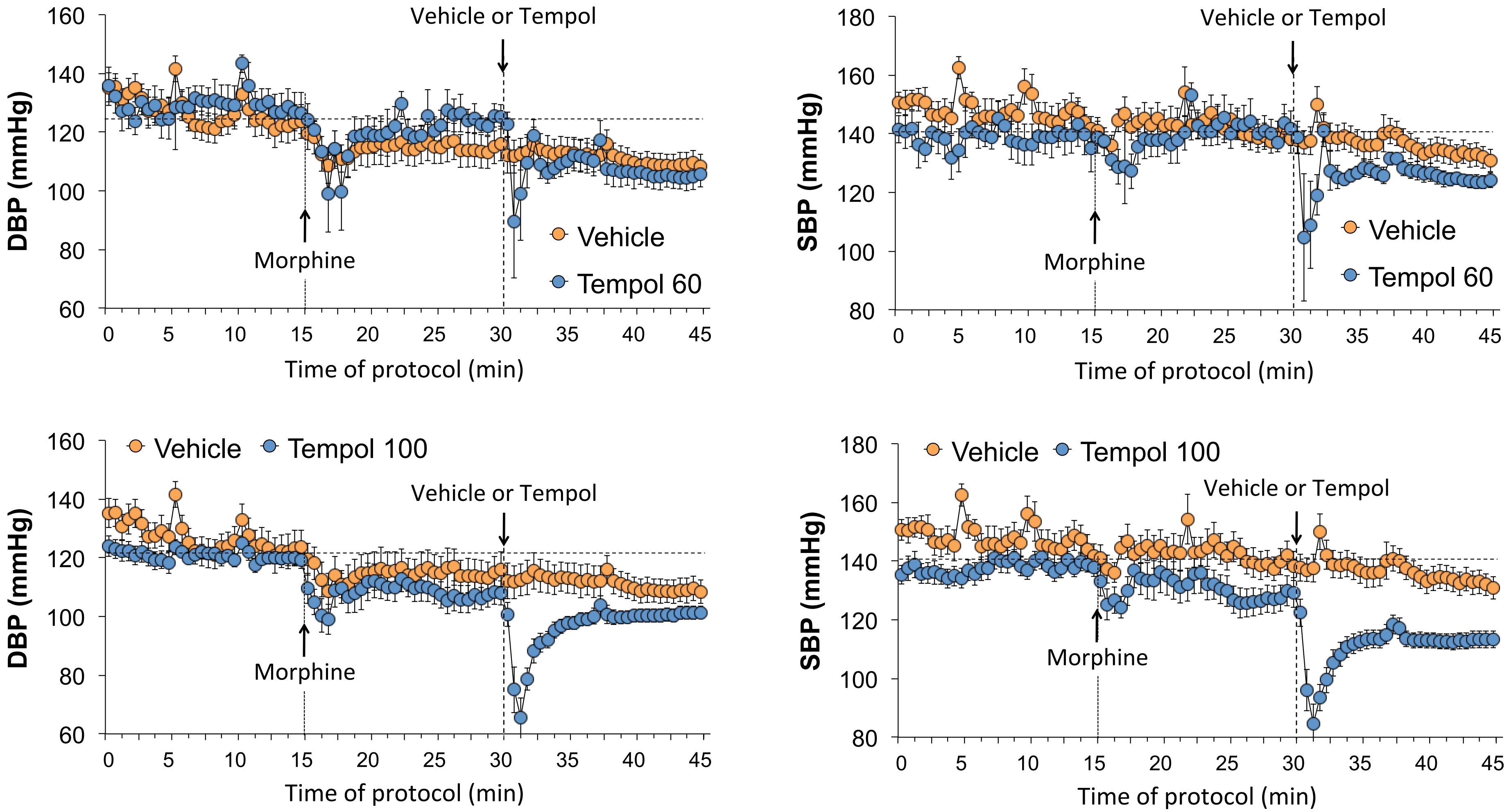

~~Supplementary~~ **Figure 1.** Changes in diastolic arterial blood pressure (DBP) and systolic arterial blood pressure (SBP) elicited by bolus injection of morphine (10 mg/kg, IV) and the subsequent injection of vehicle (saline), Tempol at 60 mg/kg, IV (Tempol 60) or Tempol at 100 mg/kg, IV (Tempol 100) in freely-moving rats. Data are shown as mean ± SEM. The numbers of rats in vehicle, Tempol 60 and Tempol 100 groups were 9, 3 and 10, respectively.

Supplemental Figure 2

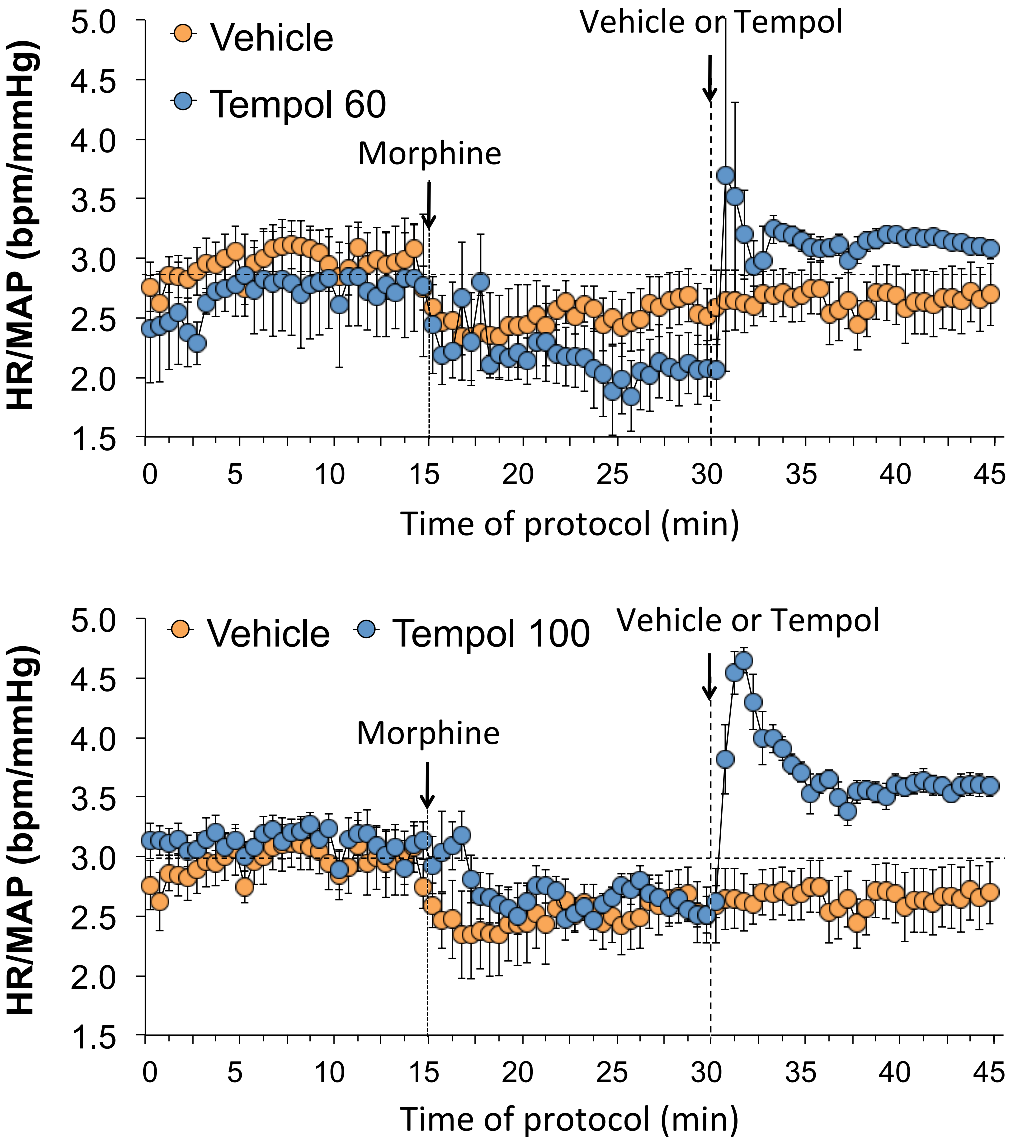

~~**Supplementary**~~ **Figure 2.** Changes in mean ~~arterial blood pressure/heart rate~~ ratios (HR/MAP) elicited by bolus injection of morphine (10 mg/kg, IV) and subsequent injection of vehicle (saline), Tempol at 60 mg/kg, IV (Tempol 60) or Tempol at 100 mg/kg, IV (Tempol 100) in freely-moving rats. Data are shown as mean ± SEM. The numbers of rats in vehicle, Tempol 60 and Tempol 100 groups were 9, 3 and 10, respectively.
